# Supplementary material for: Organic dry pea (Pisum sativum L.): A sustainable alternative pulse-based protein for human health
Source: PLoS One. 2023 Apr 12;18(4):e0284380. doi: 10.1371/journal.pone.0284380 (PMC10096185; doi:10.1371/journal.pone.0284380)
Supplement: Attachment — Submitted filename: PONE-D-22-26254_reviewer_VS.pdf [file pone.0284380.s002.pdf]

PLOS ONE

Organic dry pea (Pisum sativum L.): a sustainable alternative pulse-based protein for human health

--Manuscript Draft--

**Manuscript Number:**	PONE-D-22-26254
**Article Type:**	Research Article
**Full Title:**	Organic dry pea (Pisum sativum L.): a sustainable alternative pulse-based protein for human health
**Short Title:**	Organic Dry Pea Protein Quality
**Corresponding Author:**	Dil Thavarajah<br>Clemson University<br>Clemson, SC UNITED STATES
**Keywords:**	Dry pea, biofortification, organic breeding, plant-based proteins, sulfur-containing amino acids, lysine
**Abstract:**	Dry pea (Pisum sativum L.) is a cool-season food legume that is rich in protein (20-25%). With increasing health and ecosystem awareness, organic plant-based protein demand has increased; however, the protein quality of organic dry pea has not been well studied. This study determined the genetic variation of individual amino acids (AAs), total AAs (liberated), total protein, and in vitro protein digestibility of commercial dry pea cultivars grown in organic on-farm fields to inform the development of protein-biofortified cultivars. Twenty-five dry pea cultivars were grown in two USDA-certified organic on-farm locations in South Carolina (SC), USA, for two years. The concentrations of most individual AAs (15 of 17) and the total AA concentration significantly varied with dry pea cultivar. In vitro protein digestibility was not affected by cultivar. Total AA and protein for dry pea seeds ranged from 11.8 to 22.2 and 12.6 to 27.6 g/100 g, respectively, with heritability estimates of 0.19 to 0.25. In vitro protein digestibility and protein digestibility corrected AA score (PDCAAS) ranged from 83 to 95% and 18 to 64, respectively. Heritability estimates for individual AAs ranged from 0.08 to 0.42; principal component (PCA) analysis showed five significant AA clusters. Cultivar 'Fiddle' had significantly higher total AA (19.6 g/100 g) and digestibility (88.5%) than all other cultivars. CDC Amarillo and Jetset were significantly higher in cystine (Cys) and CDC Inca and CDC Striker were significantly higher in methionine (Met) than other cultivars; CDC Spectrum was the best option in terms of high levels of both Cys and Met. Lysine (Lys) concentration did not vary with cultivar. A 100 g serving of organic dry pea provides a significant portion of the recommended daily allowance of six essential AAs (14-189%) and daily protein (22-48%) for an average adult weighing 72 kg. Overall, this study shows organic dry pea has excellent protein quality, significant amounts of sulfur-containing AAs and Lys, and good protein digestibility, and thus has good potential for future plant-based food production. Further genetic studies are warranted with genetically diverse panels to identify candidate genes and target parents to develop nutritionally superior cultivars for organic protein production.
**Order of Authors:**	Dil Thavarajah, PHD
	Tristan Lawrence, BS
	J Lucas Boatwright, PHD
	Nathan Windsor, Undergrad
	Nathan Johnson, PHD
	Joshua Kay, BS
	Emerson Shipe, PhD
	Shiv Kumar, PHD
	Pushparajah Thavarajah, PHD
**Additional Information:**	

Powered by Editorial Manager® and ProduXion Manager® from Aries Systems Corporation

Question	Response
**Financial Disclosure**<br><br>Enter a financial disclosure statement that describes the sources of funding for the work included in this submission. Review the submission guidelines for detailed requirements. View published research articles from *PLOS ONE* for specific examples.<br><br>This statement is required for submission and **will appear in the published article** if the submission is accepted. Please make sure it is accurate.<br><br>**Unfunded studies**<br>Enter: *The author(s) received no specific funding for this work.*<br><br>**Funded studies**<br>Enter a statement with the following details:<br>• Initials of the authors who received each award<br>• Grant numbers awarded to each author<br>• The full name of each funder<br>• URL of each funder website<br>• Did the sponsors or funders play any role in the study design, data collection and analysis, decision to publish, or preparation of the manuscript?<br>• **NO** - Include this sentence at the end of your statement: *The funders had no role in study design, data collection and analysis, decision to publish, or preparation of the manuscript.*<br>• **YES** - Specify the role(s) played.<br><br>* typeset	The funders had no role in study design, data collection and analysis, decision to publish, or preparation of the manuscript.
**Competing Interests**<br><br>Use the instructions below to enter a competing interest statement for this submission. On behalf of all authors, disclose any competing interests that could be perceived to bias this work—acknowledging all financial support and any other relevant financial or non-financial competing interests.	The authors have declared that no competing interests exist.

This statement is required for submission and **will appear in the published article** if the submission is accepted. Please make sure it is accurate and that any funding sources listed in your Funding Information later in the submission form are also declared in your Financial Disclosure statement.<br><br>View published research articles from *PLOS ONE* for specific examples.<br><br>**NO authors have competing interests**<br><br>Enter: *The authors have declared that no competing interests exist.*<br><br>**Authors with competing interests**<br><br>Enter competing interest details beginning with this statement:<br><br>*I have read the journal's policy and the authors of this manuscript have the following competing interests: [insert competing interests here]*<br><br>* typeset	
**Ethics Statement**<br><br>Enter an ethics statement for this submission. This statement is required if the study involved:<br><br>• Human participants<br>• Human specimens or tissue<br>• Vertebrate animals or cephalopods<br>• Vertebrate embryos or tissues<br>• Field research<br><br>Write "N/A" if the submission does not require an ethics statement.<br><br>General guidance is provided below. Consult the submission guidelines for detailed instructions. **Make sure that all information entered here is included in the Methods section of the manuscript.**	N/A-the submission does not require an ethics statement

**Format for specific study types**<br><br>**Human Subject Research (involving human participants and/or tissue)**<br>• Give the name of the institutional review board or ethics committee that approved the study<br>• Include the approval number and/or a statement indicating approval of this research<br>• Indicate the form of consent obtained (written/oral) or the reason that consent was not obtained (e.g. the data were analyzed anonymously)<br><br>**Animal Research (involving vertebrate animals, embryos or tissues)**<br>• Provide the name of the Institutional Animal Care and Use Committee (IACUC) or other relevant ethics board that reviewed the study protocol, and indicate whether they approved this research or granted a formal waiver of ethical approval<br>• Include an approval number if one was obtained<br>• If the study involved *non-human primates*, add *additional details* about animal welfare and steps taken to ameliorate suffering<br>• If anesthesia, euthanasia, or any kind of animal sacrifice is part of the study, include briefly which substances and/or methods were applied<br><br>**Field Research**<br><br>Include the following details if this study involves the collection of plant, animal, or other materials from a natural setting:<br>• Field permit number<br>• Name of the institution or relevant body that granted permission	
**Data Availability**<br><br>Authors are required to make all data underlying the findings described fully available, without restriction, and from the time of publication. PLOS allows rare exceptions to address legal and ethical concerns. See the PLOS Data Policy and FAQ for detailed information.	Yes - all data are fully available without restriction

A Data Availability Statement describing where the data can be found is required at submission. Your answers to this question constitute the Data Availability Statement and **will be published in the article**, if accepted.<br><br>**Important:** Stating 'data available on request from the author' is not sufficient. If your data are only available upon request, select 'No' for the first question and explain your exceptional situation in the text box.<br><br>Do the authors confirm that all data underlying the findings described in their manuscript are fully available without restriction?	
**Describe where the data may be found in full sentences. If you are copying our sample text, replace any instances of XXX with the appropriate details.**<br><br>• If the data are **held or will be held in a public repository**, include URLs, accession numbers or DOIs. If this information will only be available after acceptance, indicate this by ticking the box below. For example: *All XXX files are available from the XXX database (accession number(s) XXX, XXX.*).<br>• If the data are all contained **within the manuscript and/or Supporting Information files**, enter the following: *All relevant data are within the manuscript and its Supporting Information files.*<br>• If neither of these applies but you are able to provide **details of access elsewhere**, with or without limitations, please do so. For example:<br><br>*Data cannot be shared publicly because of [XXX]. Data are available from the XXX Institutional Data Access / Ethics Committee (contact via XXX) for researchers who meet the criteria for access to confidential data.*<br><br>*The data underlying the results presented in the study are available*	All relevant data are within the manuscript and its Supporting Information files.

from (include the name of the third party and contact information or URL).<br>• This text is appropriate if the data are owned by a third party and authors do not have permission to share the data.<br><br>* typeset	
Additional data availability information:	Tick here if the URLs/accession numbers/DOIs will be available only after acceptance of the manuscript for publication so that we can ensure their inclusion before publication.

Powered by Editorial Manager® and ProduXion Manager® from Aries Systems Corporation

manuscript

Click here to access/download;Manuscript;Thavarajah - PLOSONE-9-20-2022.docx

Running Title: Organic Dry Pea Protein Quality

Organic dry pea (*Pisum sativum* L.): a sustainable alternative pulse-based protein for human health

Dil Thavarajah[*1], Tristan Lawrence[1], Lucas Boatwright[1], Nathan Windsor[1], Nathan Johnson[1], Joshua Kay[1], Emerson Shipe[1], Shiv Kumar[2], Pushparajah Thavarajah[1]

[1]Plant and Environmental Sciences, Pulse Quality and Nutritional Breeding, Biosystems Research Complex, 105 Collings St, Clemson University, Clemson, South Carolina, 29634, USA.

[2]Food Legumes Research Program, International Centre for Agricultural Research in the Dry Areas (ICARDA), Amlaha, India.

*Corresponding author

Email: dthavar@clemson.edu (DT)

Abstract

Dry pea (*Pisum sativum* L.) is a cool-season food legume that is rich in protein (20-25%). With increasing health and ecosystem awareness, organic plant-based protein demand has increased; however, the protein quality of organic dry pea has not been well studied. This study determined the genetic variation of individual amino acids (AAs), total AAs (liberated), total protein, and *in vitro* protein digestibility of commercial dry pea cultivars grown in organic on-farm fields to inform the development of protein-biofortified cultivars. Twenty-five dry pea cultivars were grown in two USDA-certified organic on-farm locations in South Carolina (SC), USA, for two years. The concentrations of most individual AAs (15 of 17) and the total AA concentration significantly varied with dry pea cultivar. *In vitro* protein digestibility was not affected by cultivar. Total AA and protein for dry pea seeds ranged from 11.8 to 22.2 and 12.6 to 27.6 g/100 g, respectively, with heritability estimates of 0.19 to 0.25. *In vitro* protein digestibility and protein digestibility corrected AA score (PDCAAS) ranged from 83 to 95% and 18 to 64, respectively. Heritability estimates for individual AAs ranged from 0.08 to 0.42; principal component (PCA) analysis showed five significant AA clusters. Cultivar 'Fiddle' had significantly higher total AA (19.6 g/100 g) and digestibility (88.5%) than all other cultivars. CDC Amarillo and Jetset were significantly higher in cystine (Cys) and CDC Inca and CDC Striker were significantly higher in methionine (Met) than other cultivars; CDC Spectrum was the best option in terms of high levels of both Cys and Met. Lysine (Lys) concentration did not vary with cultivar. A 100 g serving of organic dry pea provides a significant portion of the recommended daily allowance of six essential AAs (14-189%) and daily protein (22-48%) for an average adult weighing 72 kg. Overall, this study shows organic dry pea has excellent protein quality, significant amounts of sulfur-containing AAs and Lys, and good protein digestibility, and thus has good potential for future plant-based food production. Further genetic studies are warranted with genetically diverse panels to identify candidate genes and target parents to develop nutritionally superior cultivars for organic protein production.

Keywords: Dry pea, biofortification, organic breeding, plant-based proteins, sulfur-containing amino acids, lysine

Introduction

The plant-based protein market has been steadily growing globally. American retail sales increased by 6% in 2021, bringing the plant-based protein market's total value to $7.4B in 2021 [1]. About 39% of Americans consume plant-based protein alternative foods due to various health and ecosystem concerns [2,3]. Globally, the plant-based protein market will continue to increase to a $9.5B industry by 2025. To meet the global demand for plant-based protein, ingredient suppliers have expanded the need for novel clean, pesticide-free, and gluten-free plant proteins from organic pulse crops, including dry pea (*Pisum sativum* L.). Dry peas are the most in-demand ingredient for this segment of the food industry due to their high protein (20-25%) and low fat (<1%) levels. Certified organic dry pea production has increased in US regions that have not been historically used to grow pulse crops, including South Carolina (SC), to meet the demand for plant-based protein [4]. Current food choices including the "*Beyond Burger*" and "*Impossible Burger*" use dry pea as their primary protein ingredient rather than soy protein. However, concerns related to plant-based proteins focus on amino acid (AA) balance, i.e., legume-based protein is low in sulfur-containing AAs (SAAs: cystine, Cys and methionine, Met), and poor digestibility. The development and selection of nutritionally superior organic dry pea cultivars will bring significant economic benefits to organic growers and nutritional value to consumers.

Organic agriculture is the fastest-growing segment of US agriculture, with total sales of $9.9B in 2019 [5]. Organic grains, including corn, wheat, and soybean, accounted for $1.18B of this total, an increase of 55% from 2016 [5]. Pulse crops are an integral part of the global food system and can provide protein, low-digestible carbohydrates, and micronutrient-rich foods at a lower price than systems centered on animal proteins [4,6–8]. Certified organic dry pea production in the USA is small, with 16,666 ac in 2019 and a $5.9M value of sales from 104 USDA-certified organic farms [5]. Legume-inclusive cropping systems bring multiple benefits to organic agriculture: (1) at the food system level, pulses provide a significant amount of protein, low-digestible carbohydrates, and a range of micronutrients with low phytate for both humans and animals; (2) at the production system level, legumes fix atmospheric N and improve soil phosphorus (P), which provides economic value to organic producers, making them more suitable for low-input cropping systems and mitigating greenhouse gas emissions as a result of no N and P fertilizer inputs; and (3) at the cropping system level, legumes can be used as diversification crops in agroecosystems (e.g., in rotation with cereals), resulting in increased cereal crop yields due to

disrupted pest (disease, insect, and weed) cycles, conserved soil moisture, and improved soil health via soil microbial activity [4,9–11].

Plant-based protein represents about 60% of the total global protein consumed, with the remainder from animal sources [12]. Developing countries mainly depend on plant-based protein, and the global population is overall shifting toward plant-based alternative proteins. However, the quantity of protein in plant-based foods is not a good indicator of their ability to meet the nutritional demands of growing populations; protein quality, i.e., AA balance, must be considered [13]. Animal and plant-based proteins have different AA profiles and digestibility. Humans cannot synthesize essential AAs; therefore, these must be obtained from dietary sources [14]. Cereals have low lysine (Lys) concentrations and moderate-to-high concentrations of the SAAs, namely methionine (Met) and cysteine (Cys); however, pulses have high Lys and low-to-moderate levels of Met and Cys. Therefore, plant-based diets require that cereals be supplemented with Lys-rich ingredients such as dry pea [8,15]. With the increasing global population, dependence on animal sources for daily human protein requirements is not a viable option (e.g., higher energy and labor requirements, antibiotic resistance, and greenhouse gas emissions). Therefore, breeding traditional pulse crops for protein biofortification is essential to provide clean, allergen- and gluten-free, and highly nutritious plant-based protein to meet the world's protein demands by 2050 [8,15,16].

Biofortification is an approach to increasing nutritional quality using conventional plant breeding and genomic tools to develop staple food crops with bioavailable micronutrients [17–19]. Dry pea micronutrient enrichment has been successful over the years, and most breeding programs around the world use available tools to develop mineral- and vitamin-rich cultivars with low phytic acid [20–22]. These biofortified cultivars perform well under non-organic growing systems but have low yields and protein content when grown under an organic system without synthetic fertilizer and pesticides. Therefore, organic nutritional breeding of pulse crops for increased protein quality is vital to overcome the issues related to growers, the food industry, and the nutrition community to meet increasing consumer demand. This study aimed to evaluate if the current dry pea cultivars in production vary in protein quality (AA composition, total AA, total protein, and *in vitro* protein digestibility) in response to organic cropping systems[illegible]The objectives of this study were to assess 25 dry pea cultivars grown in two organic on-farm locations for two years to determine the genetic variation of AA profiles, total AA, protein, and *in vitro* digestibility to identify suitable cultivars for organic production with increased nutrition quality.

Materials and Methods

***Materials*:** Reagents, solvents, and high-purity standards for AA analysis were purchased from Sigma Aldrich Co. (St. Louis, MO), Fisher Scientific (Waltham, MA), and VWR International (Radnor, PA). Ultrapure water and deionized water (ddH_2O) to a resistance of ≥18.2 MΩ×cm (PURELAB flex 2 system, ELGA LabWater North America, Woodridge, IL) were used.

***Experimental details*:** The experimental field design was a randomized complete block design (RCDB) with 25 cultivars with two replications at two locations in 2019 and three replications at one location in 2020 (n=175; **Table 1)** [4]. The seeds were purchased from Pulse USA (Bismark, ND, USA), Meridian Seeds (Mapleton, ND, USA), and the Washington State Crop Improvement Association (Pullman, WA, USA). Material transfer agreements (MTAs) were signed with the seed companies before planting these cultivars in SC, USA. Detailed experimental design, agronomic details, and results (grain yield and nutritional quality) have already been published [4]. USDA-certified organic on-farm locations were WP Rawl and Sons (Pelion, SC, USA) and Calhoun Fields Laboratory (Clemson University, SC, USA). Before planting, fields were tilled using a disc harrow and smoothly leveled. Plot size was 1.4×6 m (8.4 m^2) with seven rows spaced 20 cm apart, a seeding depth of 5-7 cm, and a seeding rate of 90 seeds m^{-2}. USDA-certified organic inoculant (Peaceful Valley Farm Supply, Inc., USA) was added at 3.1 g kg^{-1} seeds. At physiological maturity (110-115 days after planting), the plots were harvested, and 500 g of seeds were hand cleaned, finely ground using a UDY grinder, and stored at −10 °C until protein quality analysis. All protein quality data are reported on a dry mass basis (15% moisture).

***Protein analysis*:** Total seed N concentration was measured using N combustion at the Soil Testing Laboratory, Clemson University, SC, and then values converted to total protein content by multiplying by 6.25. Protein data are reported in our previous publication [4].

***Amino acid (AA) analysis*:** The AA analysis is reported elsewhere [23] with modifications from the literature [24,25]. Samples (40 mg) of dry pea powder (particle size ≤ 0.5 mm) were weighed into glass culture tubes (16×125 mm, PTFE lined cap). Performic acid was synthesized from formic acid and hydrogen peroxide (9:1 ratio). Once chilled in an ice bath, 5 mL of performic acid were added to each tube, which were then gently swirled on a vortex mixer before being capped and refrigerated for 16 h to convert Cys and Met to their derivatives, methionine sulfone and cysteic acid, which are more stable under acid hydrolysis. A 1/8 in. × tube length PTFE boiling rod was inserted into each tube before evaporating to dryness in a vacuum oil bath (3 gal. resin

trap, BACOENG, Suzhou, China) at ~70–80 °C and ~610 mmHg. Once cooled, tubes were removed, and 4.9 mL of 6 M HCl and 0.1 mL of the standard internal mix (25 mM norvaline, 25 mM sarcosine) were added to each tube, which were then capped and gently swirled. Tubes were then placed in a gravity convection oven at 110 °C for 24 h to hydrolyze peptide bonds. Samples were cooled to room temperature, vortex mixed, and filtered through a 0.22 µm polypropylene syringe filter. As before, one mL of sample was added to a clean culture tube and evaporated to dryness. Samples were rehydrated with 1 mL of HPLC mobile phase A and pipetted into HPLC vials for analysis.

AA analysis was performed via high-performance reverse phase chromatography on an 1100 series Agilent system (Agilent Technologies, Santa Clara, CA, USA) [26,27] with a diode array detector at two wavelengths (338 nm, 10 nm bandwidth, reference 390 nm, 20 nm bandwidth; and 262 nm, 10 nm bandwidth, reference 390 nm, 20 nm bandwidth). An aqueous and an organic solvent were used for mobile phases A and B, respectively. Mobile phase A contained 10 mM sodium phosphate, 10 mM sodium tetraborate decahydrate, and 5 mM sodium azide with a pH adjusted to 8.2 with 12 M HCl. The solution was then filtered through 0.2 µm regenerated cellulose. Mobile phase B consisted of 45% methanol, 45% acetonitrile, and 10% water (v/v/v). A lab reference dry pea sample was included in every digestion batch to monitor batch-to-batch variation, and an AA standard mix was run on the high-performance liquid chromatograph (HPLC) before analyzing each batch of samples. Calibration standards (9–900 pmol/µL) with internal standards norvaline and sarcosine (500 pmol/µL) were run, and linear calibration models were generated based on peak areas for calculating sample AA concentrations, which were converted into percent of dry pea flour. The total AA concentration was calculated by summing all AA concentrations for each sample.

***In vitro protein digestibility analysis*:** Protein digestibility was measured using the Megazyme Protein Digestibility Amino Acid Score assay kit with the modified protocol for a 100 mg sample size (Megazyme 2019). Ground samples (100 mg) were weighed into 50 mL plastic falcon tubes, to which 3.8 mL of 0.06 N hydrochloric acid were added and the mixture vortexed. The tubes were then placed into a tabletop heated air shaker at 37 ℃ for 30 min at 300 rpm. After shaking, 0.2 mL of pepsin solution were added to the tube and the mixture vortexed. The tubes were then placed back into the shaker at 37 ℃ for 60 min at 300 rpm and, after the pepsin incubation, 0.4 mL of TRIS buffer were added. The tubes were then vortexed, and 40 µL of trypsin/chymotrypsin

solution were added with the tubes then placed back in the air shaker for 4 h. After the trypsin/chymotrypsin incubation, the tubes were placed in a 100 °C water bath for 10 min and then vortexed and brought to room temperature on the counter for a minimum of 20 min. After the overnight cold incubation, the tubes were centrifuged for 10 min. Ninety-six well plates were utilized for the colorimetric analysis. The Megazyme Excel calculator was modified to change the approximate sample mass from 0.5 to 0.1 g. In addition to the controls in the assay kit, a lab reference lentil sample was included in every batch to monitor batch-to-batch variation. The protein digestibility corrected amino acid score (PDCAAS) was calculated based on the Megazye Excel calculator, determined by comparing the AA profile of the dry pea against a standard AA profile, with 100 as the highest possible score.

Statistical analysis**:** Replicates, years, and cultivars were used as class variables. Data from both years were combined (after testing for heterogeneity) and analyzed using a general linear model procedure (PROC GLM) mixed model. Fisher's least significant difference (LSD) at ≤ 0.05 was performed for mean separation. Correlations (Pearson correlation coefficients) among traits were determined. A statistical model was developed to estimate broad-sense heritability (H^2) with the class variables and genotype as random effects. The model was fit using restricted maximum likelihood (REML). H^2 was estimated as the proportion of variance due to cultivar, and analyses were performed using JMP 14.0.0 and SAS 9.4 [28]. Percent recommended dietary allowance estimates were calculated for the essential AAs [Cys, histidine (His), isoleucine (Iso), leucine (Leu), Lys, Met, phenylalanine (Phe), threonine (Thr), valine (Val)] and total AA concentration. Estimates were based on a 72 kg adult consuming 100 g of dry pea (15% moisture content) per day: 8–12 mg/kg His, 10 mg/kg Iso, 14 mg/kg Leu, 12 mg/kg Lys, 13 mg/kg Met + Cys, 14 mg/kg Phe + Tyr, 10 mg/kg Val, and 0.8 g/kg protein [29].

Results

Analysis of variance: Cultivars showed significant variation at $P<0.05$ and $P<0.1$ for most traits except for His, hydroxyproline (Hpr), Lys, and *in vitro* protein digestibility **(Table 2)**. Location was significant for most cases except for serine (Ser) and total AAs. Similarly, the year effect was significant at $P<0.05$ and $P<0.1$ for 12 of 17 AAs, total AAs, total protein, and *in vitro* digestibility. Significant interactions of either cultivar × location or cultivar × year varied with the traits. The *in vitro* protein digestibility showed a significant effect only with the location and year; no effect was evident with cultivar × location or cultivar × year **(Table 2)**. Broad-sense heritability

estimates were very low to moderate (0.06-0.42), with the highest for arginine (Arg; 0.42) and total protein (0.25). Broad-sense heritability estimates were very low for SAAs (Met and Cys) and Lys **(Table 2)**.

Protein quality: Organic dry pea cultivars had values of 11.8 to 22.2 g/100 g for total AAs (liberated), 12.6 to 27.6 g/100 g for total protein, 18 to 64 for PDCAAS value, and 83 to 95% for *in vitro* protein digestibility **(Table 3)**. Dry pea contained a range of individual AAs, including nine essential AAs with a mean of 0.22 g/100 g for SAAs and 0.88 g/100 g for Lys **(Table 3)**. These organic dry pea cultivars provide a significant amount of the recommended daily allowance (%RDA) of several AAs (14-66% His, 79-138% Iso, 76-169% Leu, 57-147% Lys, 15-85% Met + Cys, 76-189% Phe + Tyr, 94-169% Val) as well as protein (22-48%) **(Table 3)**. Pearson's correlation analysis revealed that most correlations were significantly positive except for Hpr vs. His and *in vitro* protein digestibility vs. Lys **(Table 4)**. Total protein showed a significant positive correlation with all AAs except Hpr; Lys and Cys were also not correlated; and Hpr showed non-significant correlations in several cases **(Table 4)**. The first two principal components (PCA) of the principal component analysis (PCA) accounted for 12.46, and 1.83 for the eigenvalues. Cluster summary showed components of the total variance: (1) component 1 (62.3%): total AAs and 13 of 17 AAs; (2) component 2 (9.17%): Hpr and His; (3) component 3 (8.07%): *in vitro* protein digestibility; (4) component 4 (5.34%); protein and Arg; and (5) component 5 (2.93%): Cys **(Fig. 1)**. Most of the variation was captured by the first component (62.3%), which is highly correlated with the values of most AAs excluding Hpr and His.

Cultivar responses: Dry pea cultivars showed a normal distribution pattern for Cys, Met, total AAs, and *in vitro* protein digestibility **(Fig. 2)**. Out of 175 observations, 6.4% were high in Cys and Met, 8.8% were high in total AAs, and 5.6% were high for *in vitro* protein digestibility **(Fig. 2)**. Among the 25 cultivars tested, 10 cultivars showed more than 18 g/100 g of total AAs, with Fiddle being the highest and AAC Carver and AC Earlystar the lowest **(Fig. 3)**. For *in vitro* protein digestibility, 17 of 25 cultivars showed a digestibility of 87% or better, with Fiddle having the highest value and AAC Carver the lowest **(Fig. 3)**. CDC Saffron, CDC Spectrum, and CDC Striker showed significantly higher concentrations of SAAs than AAC Carver and AC Earlystar **(Fig. 4)**. AAC Comfort showed higher Lys concentrations than other cultivars, but the effects were not significant **(Fig. 4)**.

Discussion

Our results demonstrate that current dry pea cultivars bred for conventional systems vary in terms of seed AA profile, total AAs, total protein, and *in vitro* protein digestibility when grown under organic cropping systems. Organic dry pea is a rich source of essential AAs, as a 100 g serving of organic dry pea provides 0.02-3.07 g/100 g of nine essential AAs (14-180% of RDA), 11.8-22.2 g of total AAs, and 22-48% of the daily protein requirement, with an *in vitro* protein digestibility of 83-95% **(Table 3)**. In contrast to previous literature that states pulses are generally low in SAAs, our results demonstrate organic dry pea is a good source of SAAs (Met and Cys), with a 100 g serving providing 220 mg of total SAAs (Met+Cys) and 1.33 g of Lys **(Table 3; Fig. 4)** [30,31]. According to our knowledge, this study is the first report on the detailed protein quality of commercial dry pea cultivars grown in an organic system towards protein biofortification

The organic dry pea cultivars in this study had mean protein and total AA (liberated) concentrations of 20.9 g/100 g and 17.5 g/100 g, respectively **(Table 3)**. Several dry pea cultivars had high total AAs (>18 g/100 g) and >87% *in vitro* protein digestibility **(Fig. 3)**, demonstrating they are suitable for organic plant-based protein production. Among the cultivars tested, Fiddle had the highest total AA concentrations (19.6 g/100 g), and AAC Carver (15.5 g/100 g) and AC Earlystar (16.1 g/100 g) the lowest. Our previous study on the agronomic adaptability of dry pea [4] indicated AAC Carver, Jetset, and Mystique as the highest yielding cultivars (>2000 kg/ha) and most suitable for organic production without a yield penalty compared to conventional growing systems. However, the current study indicates these three cultivars have low total AAs and *in vitro* protein digestibility **(Fig. 3)**. A negative correlation between protein quality and crop adaptability suggests further testing is needed with diverse dry pea germplasm to develop biofortified organic cultivars with better grain yield, agronomic adaptability, and protein quality for organic systems [4,8,32]. Earlier literature [30,33] indicates the AA composition of dry pea varies with cultivar and growing environment, similar to the current study's results. Further, one of these earlier studies shows dry pea has high concentrations of Arg, Leu, Lys, aspartic acid, and glutamic acid and low concentrations of His, Met, Thr, and Cys [33]. Another study compared several plant-based protein isolates for essential and non-essential AAs and found dry pea protein isolates contained only 5.9% Lys and low concentrations of Met [34]. In contrast, our study results show most modern cultivars have higher Cys, Met, and total AA concentrations and good *in vitro* protein digestibility **(Fig. 3)**. The best options to use for better protein quality are CDC Spectrum

for Met and Cys, CDC Inca and CDC Striker for Met, and CDC Amarillo and Jetset for Cys **(Fig. 4)**. These cultivars have AA values within the range of the AAs reported in the literature for conventional cropping systems [31,34]. Incorporating these cultivars into dry pea breeding programs would benefit the development of better protein quality cultivars; however, more field testing is required to understand the genetic, environmental, and management interactions. Organic agriculture management varies with respect to on-farm practices for weeds, diseases, pests, and fertilizer; therefore, breeding dry pea cultivars best suited for organic management with increased nutritional quality is challenging [35].

AAs are critical for all forms of life. Humans cannot synthesize all 20 AAs needed for protein synthesis for good health. Nine essential AAs must be obtained from the diet: Lys, Met, and Thr of the aspartate (Asp) family pathway; phenylalanine (Phe) and tryptophan (Trp) of the aromatic AAs; Val, Ile, and Leu of the branched-chain Aas (BCAAs); and His [36]. Lys, Met, Thr, and Trp levels limit the nutritional quality of plant-based foods because levels of these four AAs in plants are very low compared with those required for optimal human nutrition [8,36]. PCA analysis in the current study revealed seven essential AAs (Val, Iso, Thr, Leu, Met, Lys, and Phe) of organic dry pea in component 1, and one essential AA (His) in component 2 **(Fig. 1)**. These essential AAs are also positively correlated with total AA, protein, and *in vitro* digestibility **(Table 4),** indicating biosynthesis of these AAs could be upregulated using available genomic and biotechnology tools for early prediction of protein quality traits in breeding programs [8,36,37]. Plant-soluble Met and Lys levels might represent limiting factors for synthesizing Met- or Lys-rich proteins [37]. Expressing genes that increase Lys and Met biosynthesis in combination with genes encoding proteins rich in Lys and Met codons appears to increase the levels of Lys in transgenic corn [37]. However, these transgenic approaches are not approved in USDA-certified organic agriculture systems. Conventional breeding approaches for selecting genetic material with higher levels of AAs and protein quality using association mapping and genomic prediction tools are the only recommended methods for organic pulse breeding.

Dietary protein quality has two components: AA composition and availability. Availability is "the proportion of the dietary amino acids that are digested and absorbed in a form suitable for body protein synthesis" [38]. PDCAAS is the most common method used to determine protein availability [39]. We determined *in vitro* protein digestibility using an enzyme assay and then calculated PDCAAS based on the AA scores. This method is inexpensive and high-throughput and

can be used to screen a larger number of seed samples for breeding programs than available *in vivo* methods [40]. The PDCAAS values for organic dry pea cultivars tested in this study ranged from 18 to 64 with 83-95% *in vitro* protein digestibility. Most organic dry pea cultivars have high protein digestibility (>87%), and these values are similar to those from the literature [41]. Plant-based proteins are an inexpensive, healthy choice for many people and a vital source of daily essential AAs. These proteins have several limitations in terms of human nutrition: they often lack one or more essential AAs, they are often not fully digestible, and toxins and pesticides are concentrated during protein extraction and drying procedures. Therefore, pursuing nutritional breeding or biofortification of dry pea using an organic system approach is vital to overcome these nutritional and production issues for pulse growers and consumers. Organic nutritional breeding of pulses is challenging and demands better phenotyping and genetic resources for cultivar development. With the increasing availability of genomic resources, expanding organic pulse breeding targets to produce better quality proteins with higher digestibility will be possible in the future.

Acknowledgments

We thank the Crop Improvement Team (Clemson University), David Robb (Organic Farm, Clemson University), and Charles Wingard and Ashely Rawls from WP Rawl & Sons, Inc. for field operations; Martin Hochhalter (Meridian Seeds), Tyler Kres (Pulse USA), and the Washington State Crop Improvement Association for providing dry pea seeds; and Elizabeth Bean, Jacob Johnson, Lindsey Moroney and Richard Baker for the technical assistant for the pulse breeding program.

Funding

Funding support for this project was provided by the Organic Agriculture Research and Extension Initiative (OREI) (award no. 2018-51300-28431/proposal no. 2018-02799) and the United States Department of Agriculture, National Institute of Food and Agriculture (award no. 2021-51300-34805/proposal no. 2021-02927) (DT, LB); the USDA National Institute of Food and Agriculture, [Hatch] project [1022664] (DT); the Good Food Institute (DT); and FoodShot Global (DT).

Funding Statement: "The funders had no role in study design, data collection, and analysis, decision to publish, or preparation of the manuscript. Its contents are solely the responsibility of the authors and do not necessarily represent the official views of the USDA." Authors have no conflict of Interest.

References

1. The Good Food Institute. 2021 State of the industry report. 2021.
2. Kerry. Winning with Plant-Based. Unlocking the Keys to Success for a Growing Market, Proprietary consumer research. 2020. https://www.fooddive.com/press-release/20200309-kerry-releases-report-on-plant-based-food-beverage-1/
3. Milo Ohr L. Plant-based protein market grows stronger. Food Technology Magazine. 2020; 74(10). https://www.ift.org/news-and-publications/food-technology-magazine/issues/2020/october/columns/nutraceuticals-plant-based-protein-market-grows-stronger
4. Thavarajah D, Lawrence TJ, Powers SE, Kay J, Thavarajah P, Shipe E, et al. Organic dry pea (Pisum sativum L.) biofortification for better human health. PLoS One. 2022; 17: e0261109. https://doi.org/10.1371/journal.pone.0261109
5. USDA -NASS. National Agriculture Statistical Services. In: 2020 [Internet]. 2017. https://quickstats.nass.usda.gov/results/FA8BCA73-7E79-3DA8-96A8-68D53707599D
6. Thavarajah D, Thavarajah P, Wejesuriya A, Rutzke M, Glahn RP, Combs Jr. GF, et al. The potential of lentil (Lens culinaris L.) as a whole food for increased selenium, iron, and zinc intake: Preliminary results from a 3 year study. Euphytica. 2011; 180. doi:10.1007/s10681-011-0365-6
7. Johnson N, Johnson CR, Thavarajah P, Kumar S, Thavarajah D. The roles and potential of lentil prebiotic carbohydrates in human and plant health. Plants, People, Planet. 2020; n/a. doi:10.1002/ppp3.10103
8. Salaria S, Boatwright JL, Thavarajah P, Kumar S, Thavarajah D. Protein biofortification in lentils (Lens culinaris Medik.) toward human health. Front Plant Sci. 2022; 13. doi:10.3389/fpls.2022.869713
9. Stagnari F, Maggio A, Galieni A, Pisante M. Multiple benefits of legumes for agriculture sustainability: an overview. Chemical and Biological Technologies in Agriculture. 2017: 4(2). doi:10.1186/s40538-016-0085-1
10. Powers SE, Thavarajah D. Checking agriculture's pulse: Field pea (Pisum Sativum L.), sustainability, and phosphorus use efficiency. Front Plant Sci. 2019; 10. doi:10.3389/fpls.2019.01489
11. Powers S, Mirsky E, Bandaranayake A, Thavarajah P, Shipe E, Bridges W, et al. Field pea

(Pisum sativum L.) shows genetic variation in phosphorus use efficiency in different P environments. Sci Rep. 2020; 10. doi:10.1038/s41598-020-75804-0
12. Nijdam D, Rood T, Westhoek H. The price of protein: Review of land use and carbon footprints from life cycle assessments of animal food products and their substitutes. Food Policy. 2012; 37, 760–770. https://doi.org/10.1016/j.foodpol.2012.08.002
13. Ritchie H, Reay DS, Higgins P. Beyond calories: A holistic assessment of the global food system. Frontiers in Sustainable Food Systems. 2018: 2, 57. https://www.frontiersin.org/article/10.3389/fsufs.2018.00057
14. Lizarazo CI, Lampi A-M, Liu J, Sontag-Strohm T, Piironen V, Stoddard FL. Nutritive quality and protein production from grain legumes in a boreal climate. J Sci Food Agric. 2015; 95, 2053–2064. https://doi.org/10.1002/jsfa.6920
15. Leinonen I, Iannetta PPM, Rees RM, Russell W, Watson C, Barnes AP. Lysine supply is a critical factor in achieving sustainable global protein economy. Frontiers in Sustainable Food Systems. 2019; 3, 27. https://www.frontiersin.org/article/10.3389/fsufs.2019.00027
16. Khazaei H, Subedi M, Nickerson M, Martínez-Villaluenga C, Frias J, Vandenberg A. Seed protein of lentils: Current status, progress, and food applications. Foods. 2019; 8. doi:10.3390/foods8090391
17. Bouis HE, Welch RM. Biofortification—A sustainable agricultural strategy for reducing micronutrient malnutrition in the global south. Crop Sci. 2010; 50, S-20-S-32. doi:10.2135/cropsci2009.09.0531
18. Dil T, Johnson CR, McGee R, Thavarajah P. Phenotyping nutritional and antinutritional traits. In: Kumar J, Pritap S, Kumar S (Eds.), Phenomics in Crop Plants: Trends, Options and Limitations. 2015. doi:10.1007/978-81-322-2226-2_15
19. Jha AB, Warkentin TD. Biofortification of pulse crops: Status and future perspectives. Plants. 2020;9. doi:10.3390/plants9010073
20. Thavarajah D, Warkentin T, Vandenberg A. Natural enrichment of selenium in Saskatchewan field peas (Pisum sativum L.). Can J Plant Sci. 2010; 90(4). doi:10.4141/CJPS09154
21. Ray H, Bett K, Tar'an B, Vandenberg A, Thavarajah D, Warkentin T. Mineral micronutrient content of cultivars of field pea, chickpea, common bean, and lentil grown in Saskatchewan, Canada. Crop Sci. 2014; 54. doi:10.2135/cropsci2013.08.0568

22. Shunmugam AS, Liu X, Stonehouse R, Tar'An B, Bett KE, Sharpe AG, et al. Mapping seed phytic acid concentration and iron bioavailability in a pea recombinant inbred line population. Crop Sci. 2015; 55, 828–836. doi:10.2135/cropsci2014.08.0544
23. Madurapperumage A, Johnson N, Thavarajah P, Tang L, Thavarajah D. Fourier-transform infrared spectroscopy (FTIR) as a high-throughput phenotyping tool for quantifying protein quality in pulse crops. Plant Phenome J. 2022; 5, e20047. doi:https://doi.org/10.1002/ppj2.20047
24. Gehrke CW, Wall Sr LL, Absheer JS, Kaiser FE, Zumwalt RW. Sample preparation for chromatography of amino acids: Acid hydrolysis of proteins. J Assoc Off Anal Chem. 1985; 68, 811–821. doi:10.1093/jaoac/68.5.811
25. Manneberg M, Lahm HW, Fountoulakis M. Quantification of cysteine residues following oxidation to cysteic acid in the presence of sodium azide. Anal Biochem. 1995; 231, 349–353. doi:10.1006/abio.1995.9988
26. Agilent Application Note. Separation of two sulfurated amino acids with other seventeen amino acids by HPLC with pre-column derivatization. Agilent Technol. 2010.
27. Long W. Automated Amino Acid Analysis Using an Agilent Poroshell HPH-C18 Column. Agilent Technol. 2015.
28. SAS Institute Inc. SAS Institute Inc. Cary; NC; 2013: User's guide: Statistics SAS Institute; 2013.
29. National Research Council (US) Subcommittee on the Tenth Edition of the Recommended Dietary Allowances. In: Recommended Dietary Allowances: 10th Edition. Washington (DC): National Academies Press (US) [Internet]. 1989 [cited 26 Aug 2022] p. 6. https://www.ncbi.nlm.nih.gov/books/NBK234922/
30. Khan MA, Jacobsen I, Eggum BO. Nutritive value of some improved varieties of legumes. J Sci Food Agric. 1979; 30, 395–400. https://doi.org/10.1002/jsfa.2740300409
31. Bonke A, Sieuwerts S, Petersen IL. Amino acid composition of novel plant drinks from oat, lentil and pea. Foods. 2020; 9, 429. doi:10.3390/foods9040429
32. Daba SD, Morris CF. Pea proteins: Variation, composition, genetics, and functional properties. Cereal Chem. 2022; 99, 8–20. https://doi.org/10.1002/cche.10439
33. Holt NW, Sosulski FW. Amino acid composition and protein quality of field peas. Can J Plant Sci. 1979; 59, 653–660. doi:10.4141/cjps79-103

34. Gorissen SHM, Crombag JJR, Senden JMG, Waterval WAH, Bierau J, Verdijk LB, et al. Protein content and amino acid composition of commercially available plant-based protein isolates. Amino Acids. 2018; 50, 1685–1695. doi:10.1007/s00726-018-2640-5

35. Boyhan GE, Stone SP. Breeding for organic and sustainable production. In: Nandwani D (Ed.), Organic Farming for Sustainable Agriculture. Cham: Springer International Publishing; 2016. pp. 123–136. doi:10.1007/978-3-319-26803-3_6

36. Galili G, Amir R, Fernie AR. The regulation of essential amino acid synthesis and accumulation in plants. Annu Rev Plant Biol. 2016; 67, 153–178. doi:10.1146/annurev-arplant-043015-112213

37. Galili G, Amir R. Fortifying plants with the essential amino acids lysine and methionine to improve nutritional quality. Plant Biotechnol J. 2013; 11, 211–222. https://doi.org/10.1111/pbi.12025

38. Rutherfurd SM, Moughan PJ. Available versus digestible dietary amino acids. Br J Nutr. 2012; 108, S298–S305. doi:10.1017/S0007114512002528

39. FAO/WHO. Dietary protein quality evaluation in human nutrition. Report of an FAO expert consultation. Food and Nutrition Paper No 9. 2013.

40. Cordero-Clavijo LM, Serna-Saldívar SO, Lazo-Vélez MA, González JFA-, Panata-Saquicilí D, Briones-Garcia M. Characterization, functional and biological value of protein-enriched defatted meals from sacha inchi (Plukenetia volubilis) and chocho (Lupinus mutabilis). J Food Meas Charact. 2021; 15: 5071–5077. doi:10.1007/s11694-021-01084-5

41. Nosworthy MG, Franczyk AJ, Medina G, Neufeld J, Appah P, Utioh A, et al. Effect of processing on the in vitro and in vivo protein quality of yellow and green split peas (Pisum sativum). J Agric Food Chem. 2017; 65: 7790–7796. doi:10.1021/acs.jafc.7b03597

Table 1: Experimental design used in the study.

Year (location)	2019 (Clemson; Pelion), 2020 (Pelion)
Location	Clemson, SC; Pelion SC
Replicates (Year)	2 (2019); 3 (2020)
Cultivars (25)	AAC Carver, AAC Comfort, AC Agassiz, AC Earlystar, Banjo, CDC Amarillo, CDC Greenwater, CDC Inca, CDC Saffron, CDC Spectrum, CDC Striker, Delta, DS Admiral, Durwood, Fiddle, Flute, Hampton, Jetset, Korando, LG Koda, Matrix, Mystique, Nette 2010, SW Arcadia, SW Midas
Total	175

Table 2: Analysis of variance and broad-sense heritability estimates of protein quality traits evaluated for dry pea tested in SC, USA.

Component	Cultivar	Location	Year	Cultivar × Location	Cultivar × Year	H^2
Alanine	**	**	*	**	**	0.11
Arginine	**	**	**	NS	*	0.42
Asparagine	**	**	**	**	**	0.08
Cystine	*	**	NS	**	**	-
Glutamine	**	**	NS	**	**	0.24
Glycine	**	*	**	**	**	0.19
Histidine	NS	**	NS	NS	NS	0.14
Hydroxyproline	NS	**	**	NS	NS	-
Isoleucine	**	**	**	**	**	0.23
Leucine	**	**	**	**	**	0.18
Lysine	NS	*	**	NS	NS	0.17
Methionine	**	*	NS	NS	NS	0.12
Phenylalanine	*	**	NS	**	*	0.23
Proline	**	**	**	*	**	0.18
Serine	**	NS	**	**	**	0.13
Threonine	**	*	**	**	**	0.06
Valine	**	**	**	**	**	0.13
Total AA	**	NS	**	**	**	0.19
Total Protein	**	**	**	NS	*	0.25
In-vitro Digestibility	NS	**	**	NS	NS	0.09

** significant at $P<0.05$; * significant at $P<0.1$; Not significant (NS); H^2 broad-sense heritability estimate.

Table 3: Range and mean amino acid concentrations of organic dry pea grown in SC.

Composition (g/100 g)	Range	Mean	Genotype Effect	%RDA
Alanine	0.61-1.01	0.86	**	
Arginine	0.95-2.22	1.5	**	
Asparagine	1.59-3.07	2.36	**	
Cystine	0.02-0.10	0.05	*	15-85
Glutamine	1.82 -3.56	2.86	**	
Glycine	0.60-1.08	0.88	**	
Histidine	0.08-0.38	0.26	NS	14-66
Hydroxyproline	0.48-2.00	1.16	NS	
Isoleucine	0.57-0.99	0.8	**	79-138
Leucine	0.77-1.70	1.33	**	76-169
Lysine	0.49-1.27	0.88	NS	57-147
Methionine	0.12-0.26	0.17	**	
Phenylalanine	0.38-1.16	0.89	*	76-189
Proline	0.42-1.32	1.04	**	
Serine	0.58-1.09	0.89	**	
Threonine	0.39-0.74	0.59	**	
Valine	0.68-1.22	0.97	**	
Total AA (liberated)	11.8-22.2	17.5	**	
Total Protein[±]	12.6-27.6	20.9	**	22-48
PDCAAS value	18-64	54	ND	
In vitro digestibility (%)	83-95	87	NS	

** significant at $P<0.05$; * significant at $P<0.1$; Not significant (NS); ND: Not detected; PDCAAS: Protein digestibility corrected amino acid score. [±] Protein values are from [4]. Values are based on the combined statistical analysis of 175 data points for the current study (dry weight basis). Percent recommended dietary allowance estimates were calculated for the essential amino acids cystine (Cys), histidine (His), isoleucine (Iso), leucine (Leu), lysine (Lys), methionine (Met), phenylalanine (Phe), threonine (Tyr), and valine (Val), as well as for total AA concentration. Estimates were for a 72 kg adult consuming 100 g of dry pea (15% moisture content) per day given the following dietary requirements: 8–12 mg/kg His, 10 mg/kg Iso, 14 mg/kg Leu, 12 mg/kg Lys, 13 mg/kg Met + Cys, 14 mg/kg Phe + Tyr, 10 mg/kg Val, and 0.8 g/kg protein [29].

Table 4: Pearson's correlation analysis of nutritional traits among dry pea cultivars grown in the organic system.

	Cys	Asp	Glu	Ser	His	Gly	Thr	Met	Arg	Ala	Val	Phe	Iso	Leu	Lys	Hpr	Pro	AA	Pr	Dig
Cys	-																			
Asp	**	-																		
Glu	**	**	-																	
Ser	**	**	**	-																
His	**	**	**	**	-															
Gly	**	**	**	**	**	-														
Thr	**	**	**	**	**	**	-													
Met	**	**	**	**	**	**	**	-												
Arg	**	**	**	**	**	**	**	**	-											
Ala	**	**	**	**	**	**	**	**	**	-										
Val	**	**	**	**	**	**	**	**	**	**	-									
Phe	**	**	**	**	**	**	**	**	**	**	**	-								
Iso	**	**	**	**	**	**	**	**	**	**	**	**	-							
Leu	**	**	**	**	**	**	**	**	**	**	**	**	**	-						
Lys	NS	**	**	**	**	**	**	**	**	**	**	**	**	**	-					
Hpr	NS	**	*	**	-**	**	**	**	NS	NS	**	NS	NS	**	NS	-				
Pro	**	**	**	**	**	**	**	**	**	**	**	**	**	**	**	**	-			
Total AA	**	**	**	**	**	**	**	**	**	**	**	**	**	**	**	**	**	-		
Total Protein±	**	**	**	**	**	**	**	**	**	**	**	**	**	**	**	NS	**	**	-	
Digestibility	**	**	**	**	*	**	**	**	**	**	**	**	**	**	-**	**	**	**	**	-

** significant at *P<0.05*; Not significant (NS); ± Protein values are from [4].

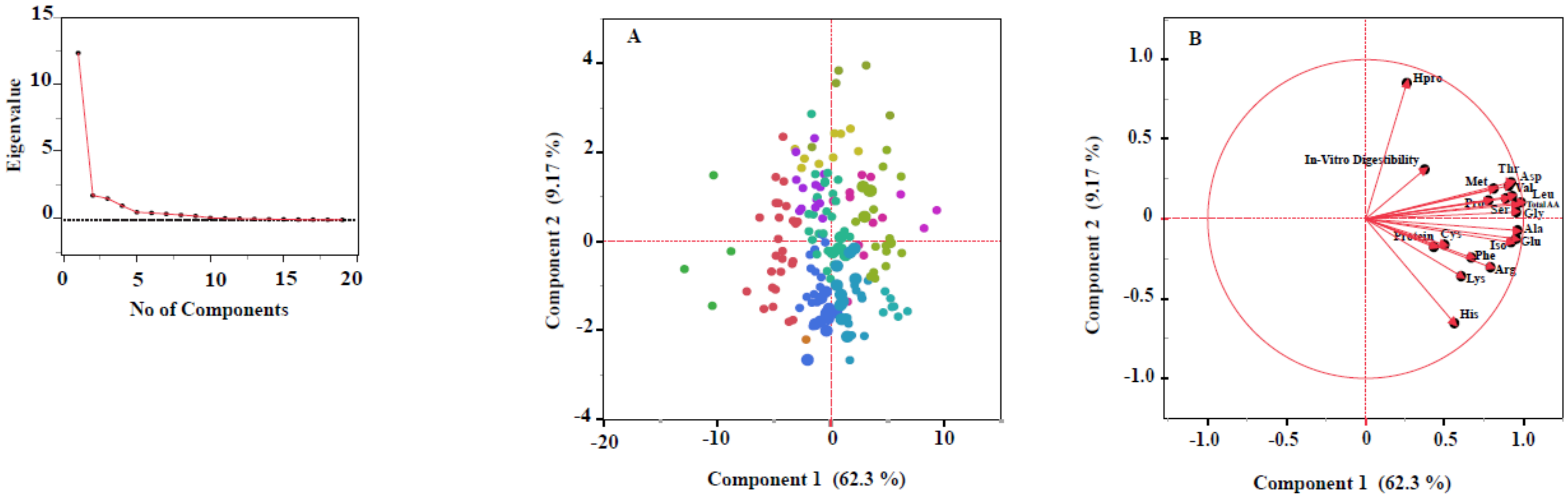

Figure 1: Principal components of individual amino acids (g/100 g), total amino acids (g/100 g), protein (g/100 g), and *in vitro* digestibility of organic dry pea: (A) scatter plots and (B) biplots of components 1 and 2. Component 1 includes total AA, valine, alanine, glycine, serine, asparagine, isoleucine, threonine, leucine, glutamine, methionine, proline, lysine, and phenylalanine; Component 2 includes hydroxyproline and histidine; Component 3 includes *in vitro* digestibility; Component 4 includes total protein and arginine; Component 5 includes cystine.

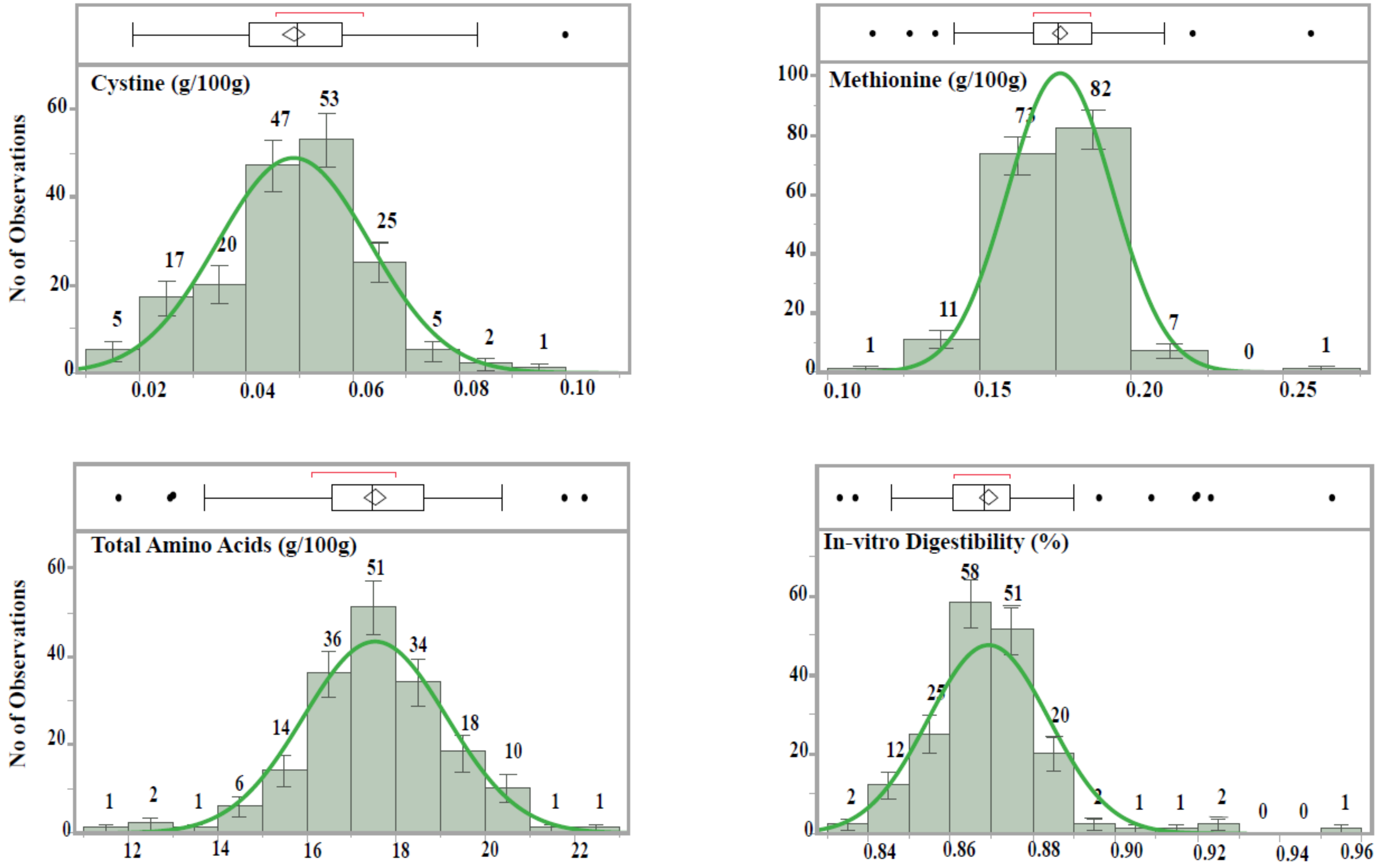

Figure 2: Dry pea cultivar distribution for cystine, methionine, and total amino acid (liberated) concentration as well as *in vitro* digestibility.

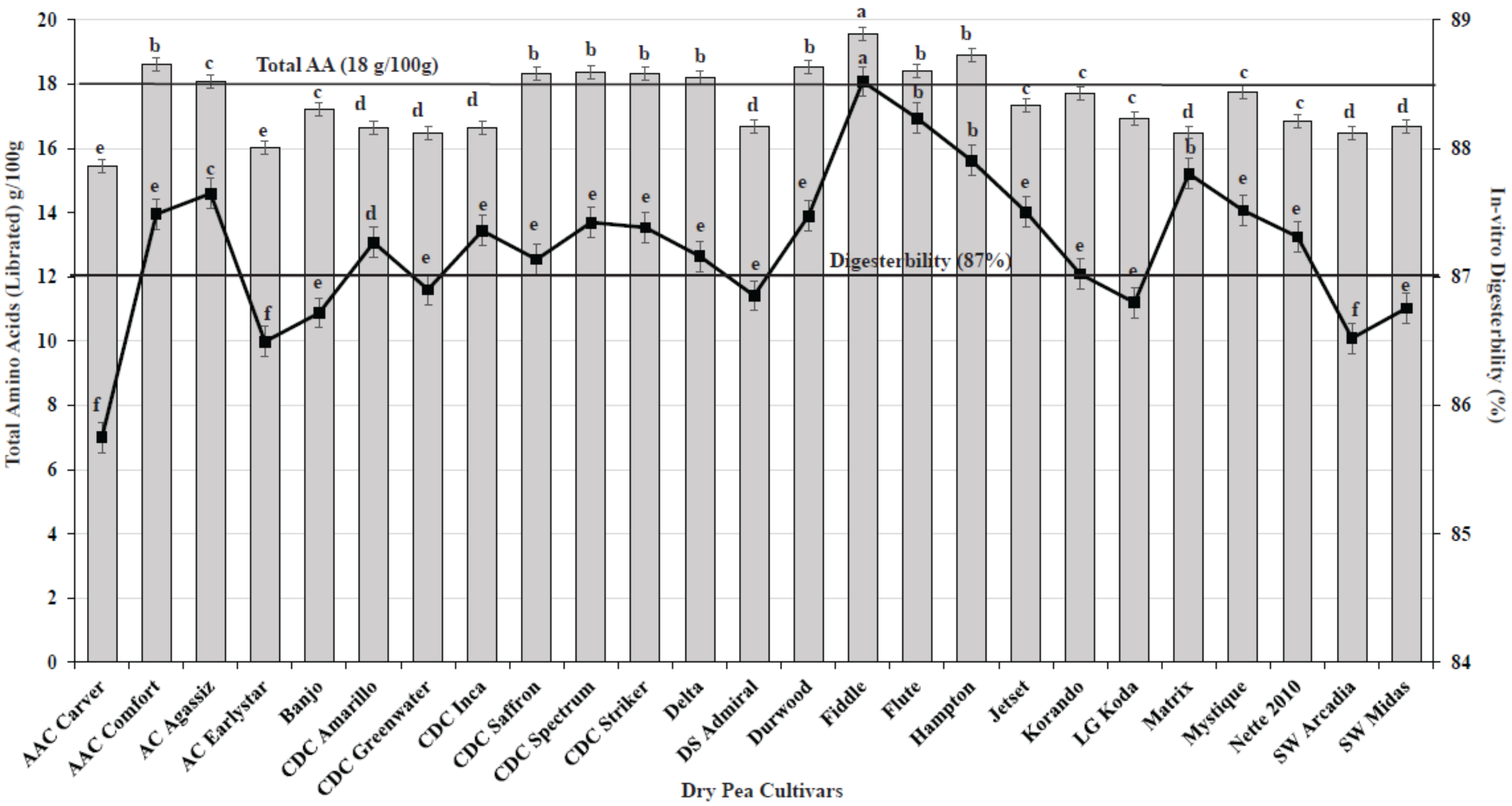

Figure 3: Total amino acids (liberated, g/100 g) and *in vitro* protein digestibility (%) of dry pea cultivars grown in the organic system

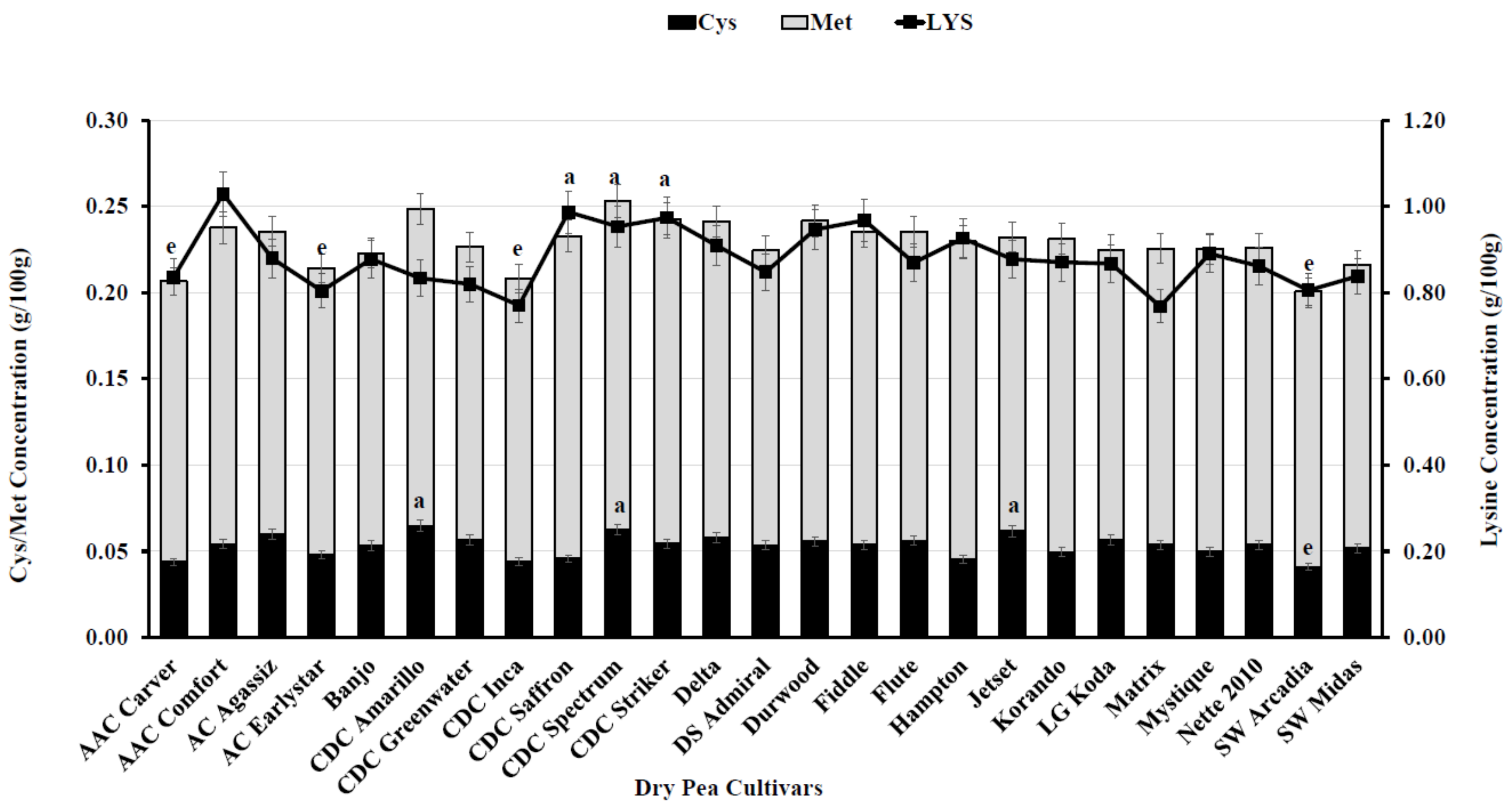

Figure 4: Organic dry pea cultivar genetic variation for seed cystine, methionine, and lysine concentrations

Click here to access/download
Supporting Information
S1-Supplementary data.xlsx
