# Supplementary material for: Organic dry pea (Pisum sativum L.): A sustainable alternative pulse-based protein for human health
Source: PLoS One. 2023 Apr 12;18(4):e0284380. doi: 10.1371/journal.pone.0284380 (PMC10096185; doi:10.1371/journal.pone.0284380)
Supplement: Attachment — Submitted filename: Reviewers comments-12-5-2022.docx [file pone.0284380.s003.docx]

**Response to Reviewers:**

Journal Requirements:

When submitting your revision, we need you to address these additional requirements.

1. Please ensure that your manuscript meets PLOS ONE's style requirements, including those for file naming. The PLOS ONE style templates can be found at 
[https://journals.plos.org/plosone/s/file?id=wjVg/PLOSOne_formatting_sample_main_body.pdf](https://urldefense.com/v3/__https:/journals.plos.org/plosone/s/file?id=wjVg*PLOSOne_formatting_sample_main_body.pdf__;Lw!!PTd7Sdtyuw!SE8F8d4DPOflhKbILH4hDzKQO9levW6GuSDgOHv54-V8YHZjU9PWajwlyuOOfFcJ90ezQaJKF1ykL6Z8RA$) and 
[https://journals.plos.org/plosone/s/file?id=ba62/PLOSOne_formatting_sample_title_authors_affiliations.pdf](https://urldefense.com/v3/__https:/journals.plos.org/plosone/s/file?id=ba62*PLOSOne_formatting_sample_title_authors_affiliations.pdf__;Lw!!PTd7Sdtyuw!SE8F8d4DPOflhKbILH4hDzKQO9levW6GuSDgOHv54-V8YHZjU9PWajwlyuOOfFcJ90ezQaJKF1y1dpJqMQ$)

**Response: Action has been taken.**

2. We note that the grant information you provided in the ‘Funding Information’ and ‘Financial Disclosure’ sections do not match.

When you resubmit, please ensure that you provide the correct grant numbers for the awards you received for your study in the ‘Funding Information’ section.

**Response: Action has been taken.**

3. Thank you for stating the following financial disclosure: 
"The funders had no role in study design, data collection and analysis, decision to publish, or preparation of the manuscript."

**At this time, please address the following queries:**

a) Please clarify the sources of funding (financial or material support) for your study. List the grants or organizations that supported your study, including funding received from your institution.

**Response:**

**Founding Agencies:**

1. National Institute of Food and Agriculture, Organic Agriculture Research and Extension Initiative (OREI) (award no. 2018-51300-28431/proposal no. 2018-02799) and the United States Department of Agriculture,
2. National Institute of Food and Agriculture, Organic Agriculture Research and Extension Initiative (OREI) (award no. 2021-51300-34805/proposal no. 2021-02927) (DT, LB)
3. USDA National Institute of Food and Agriculture, [Hatch] project [1022664] (DT);
4. Good Food Institute (DT)
5. FoodShot Global (DT)
b) State what role the funders took in the study. If the funders had no role in your study, please state:

**Response: Funders have no role in this study: The funders had no role in study design, data collection, and analysis, decision to publish, or preparation of the manuscript.**

c) If any authors received a salary from any of your funders, please state which authors and which funders.

**Response: No salary was received from funders.**
d) If you did not receive any funding for this study, please state: “The authors received no specific funding for this work.”

**Response**: No action is needed.

Please include your amended statements within your cover letter; we will change the online submission form on your behalf.

**Response: Action has been taken – all the information was added to the cover letter.**

4. We note that you have stated that you will provide repository information for your data at acceptance. Should your manuscript be accepted for publication, we will hold it until you provide the relevant accession numbers or DOIs necessary to access your data. If you wish to make changes to your Data Availability statement, please describe these changes in your cover letter and we will update your Data Availability statement to reflect the information you provide.

**Response: All data used for this manuscript is available as a supplementary file attached to the manuscript.**

5. Please include captions for your Supporting Information files at the end of your manuscript, and update any in-text citations to match accordingly. Please see our Supporting Information guidelines for more information: [http://journals.plos.org/plosone/s/supporting-information](https://urldefense.com/v3/__http:/journals.plos.org/plosone/s/supporting-information__;!!PTd7Sdtyuw!SE8F8d4DPOflhKbILH4hDzKQO9levW6GuSDgOHv54-V8YHZjU9PWajwlyuOOfFcJ90ezQaJKF1ylK9sPnQ$).

**Action taken.**

6. Please review your reference list to ensure that it is complete and correct. If you have cited papers that have been retracted, please include the rationale for doing so in the manuscript text, or remove these references and replace them with relevant current references. Any changes to the reference list should be mentioned in the rebuttal letter that accompanies your revised manuscript. If you need to cite a retracted article, indicate the article’s retracted status in the References list and also include a citation and full reference for the retraction notice.

**Action taken.**

**Additional Editor Comments:**

I am now in receipt of review reports for your manuscript. As you would notice, both find the MS to be of interest while one of them has been especially critical on some of the aspects which need to be thoroughly addressed while submitting your revised version. Beside this, I also have my own observations which require attention:
1. The topic has been introduced mostly in light of the US markets and cultivation practices while there are several other regions where dry peas are an important crop. Accordingly, the same along with supporting data needs to be mentioned in the 'Introduction' section.

**Response**: Please see the new paragraph line 66-73.

2. The source of consumables may be mentioned within the text as and when required and not as a separate paragraph, that too the first para of the 'M&M' section.

**Response**: Removed the paragraph and added it to the text.

3. While a reference has been cited (lines 129-130) about agronomic practices, etc. it is advised to add a couple of sentences on the same to give the authors a continuity in case they cannot immediately consult the previous paper.

**Response**: Please see the new lines 133-134.

4. Table 1 in not required.

**Response**: Table 1 has been removed.

[Note: HTML markup is below. Please do not edit.]

Reviewers' comments:

Reviewer's Responses to Questions

**Comments to the Author**

1. Is the manuscript technically sound, and do the data support the conclusions?

The manuscript must describe a technically sound piece of scientific research with data that supports the conclusions. Experiments must have been conducted rigorously, with appropriate controls, replication, and sample sizes. The conclusions must be drawn appropriately based on the data presented.

Reviewer #1: Partly

Reviewer #2: Yes

2. Has the statistical analysis been performed appropriately and rigorously?

Reviewer #1: No

Reviewer #2: Yes

3. Have the authors made all data underlying the findings in their manuscript fully available?

The [PLOS Data policy](https://urldefense.com/v3/__http:/www.plosone.org/static/policies.action*sharing__;Iw!!PTd7Sdtyuw!SE8F8d4DPOflhKbILH4hDzKQO9levW6GuSDgOHv54-V8YHZjU9PWajwlyuOOfFcJ90ezQaJKF1whG3qf5g$) requires authors to make all data underlying the findings described in their manuscript fully available without restriction, with rare exception (please refer to the Data Availability Statement in the manuscript PDF file). The data should be provided as part of the manuscript or its supporting information, or deposited to a public repository. For example, in addition to summary statistics, the data points behind means, medians and variance measures should be available. If there are restrictions on publicly sharing data—e.g. participant privacy or use of data from a third party—those must be specified.

Reviewer #1: Yes

Reviewer #2: Yes

4. Is the manuscript presented in an intelligible fashion and written in standard English?

PLOS ONE does not copyedit accepted manuscripts, so the language in submitted articles must be clear, correct, and unambiguous. Any typographical or grammatical errors should be corrected at revision, so please note any specific errors here.

Reviewer #1: Yes

Reviewer #2: Yes

5. Review Comments to the Author

Please use the space provided to explain your answers to the questions above. You may also include additional comments for the author, including concerns about dual publication, research ethics, or publication ethics. (Please upload your review as an attachment if it exceeds 20,000 characters)

Reviewer #1: The focus of this paper is on organic pea. The authors should speculate on whether their results would have differed had the research been conducted under conventional agriculture instead of organic. As organic pea production is a very small industry in the world, the authors should comment on whether their results could be more broadly applicable to conventional agriculture. What are the novel findings in this paper other than organic?

**Response**: Thank you for the comments. A sentence will be added to the discussion.

My specific comments:
The abstract gives the impression that the field studies were conducted over 4 station-years (2 years X 2 locations), however, in reality it was only 3 station-years. This should be made clear. Three station-years is quite minimal for field based agricultural research and this limitation should be mentioned in the paper. With only three station years conducted, I recommend that the data are reanalyzed in that way instead of trying to separate the effects of year and location since the number of years and locations was minimal.

**Response**: thank you for the response. Clarity was included in the abstract and the method section. We did not separate the year and location effect, and data were analyzed as combined (lines 190-192).

Line 38. PDCAAS is usually expressed as a number between 0 and 1.
**Response**: Thank you, it was corrected. Sorry for the typo. New lines 187-189.

63 novel,

**Response**: corrected.

66 indicate the number of acres and tonnes of organic pea production in the USA and by region in USA
**Response**: New lines 66-71. Organic dry pea acreage is not available yet.

68 Impossible burger uses soybean protein, not pea protein

**Response:** Thank you for the correction, yes it was removed.

93 is not the only indicator ...
**Response**: corrected.

107 likely not 'most breeding programs'

**Response**: corrected

109-111 provide a reference for this statement

**Response**: Reference is added.

124 only 3 station-years, so it is a limited dataset.

**Response:** Thank you for the comments. I agree that it is better to have more locations and years but with limited funding availability for our research most pre-breeding studies are conducted for two years and use selected parents for speed breeding.

142 if the AA analysis is already published, what is novel in this paper? Or do you mean that the METHOD of AA analysis is already published?

**Response**: We published the AA analysis method not the data. Please see new line 150.

142 why was tyrptophan omitted from this research?

**Response**: Tryptophan is breakdown during acid hydrolysis and concentrations will be below the detection limits. Therefore, we are not reporting tryptophan.

221 please include %RDA for all AAs, especially met.

**Response**: %RDA can be calculated only for the estimates that are available for His, Iso, Lue, Lys, Met+Cys, Phe+Tyr and Val only from reference 31. It is not able to calculate individually for the Met and Cys. Methionine values are the same as cystine values as it gives a sum of Met and Cys. Please refer to reference 31 for the RDA values that are only available for listed essential AA. Please see the footnote in the Table 2.

254 the cultivars are not 'organic', the study was conducted under organic conditions
**Response**: Corrected – thank you.

270 isolates are different from whole seeds studied here, so it is not a fair comparison
**Response**: Agree, but isolates were extracted from the whole seed. It is good information to include in the discussion.

311 'toxins and pesticides' - really?! please provide a reference for this statement.

**Response**: Yes, that is correct, as glyphosate and other pesticides are concentrated during the protein isolation process. My lab has filed a patent to develop organic proteins without harmful pesticides and chemicals. The patent will be available to the public next year. We go for organic pulse breeding to avoid pesticides and other chemicals in our pea and lentil proteins. I also include a reference.

Reviewer #2: This manuscript hasn't got enough substance/findings to be published in a reputed journal, however findings on variability in AAs and digestibility among dry pea varieties are new and informative, that's why I am recommending it to be published.

**Response**: All comments attached to the PDF was corrected.

6. PLOS authors have the option to publish the peer review history of their article ([what does this mean?](https://urldefense.com/v3/__https:/journals.plos.org/plosone/s/editorial-and-peer-review-process*loc-peer-review-history__;Iw!!PTd7Sdtyuw!SE8F8d4DPOflhKbILH4hDzKQO9levW6GuSDgOHv54-V8YHZjU9PWajwlyuOOfFcJ90ezQaJKF1y4vT2N7w$)). If published, this will include your full peer review and any attached files.


If you choose “no”, your identity will remain anonymous but your review may still be made public.


**Do you want your identity to be public for this peer review?** For information about this choice, including consent withdrawal, please see our [Privacy Policy](https://urldefense.com/v3/__https:/www.plos.org/privacy-policy__;!!PTd7Sdtyuw!SE8F8d4DPOflhKbILH4hDzKQO9levW6GuSDgOHv54-V8YHZjU9PWajwlyuOOfFcJ90ezQaJKF1zb2xtxaA$).

Reviewer #1: No

Reviewer #2: No

[NOTE: If reviewer comments were submitted as an attachment file, they will be attached to this email and accessible via the submission site. Please log into your account, locate the manuscript record, and check for the action link "View Attachments". If this link does not appear, there are no attachment files.]

While revising your submission, please upload your figure files to the Preflight Analysis and Conversion Engine (PACE) digital diagnostic tool, [https://pacev2.apexcovantage.com/](https://urldefense.com/v3/__https:/pacev2.apexcovantage.com/__;!!PTd7Sdtyuw!SE8F8d4DPOflhKbILH4hDzKQO9levW6GuSDgOHv54-V8YHZjU9PWajwlyuOOfFcJ90ezQaJKF1w-wwdNFg$). PACE helps ensure that figures meet PLOS requirements. To use PACE, you must first register as a user. Registration is free. Then, login and navigate to the UPLOAD tab, where you will find detailed instructions on how to use the tool. If you encounter any issues or have any questions when using PACE, please email PLOS at [figures@plos.org](mailto:figures@plos.org). Please note that Supporting Information files do not need this step.
